# A Case of Prostate Cancer Presenting with Rash

**DOI:** 10.7759/cureus.4734

**Published:** 2019-05-23

**Authors:** Anas Albawaliz, Waled Bahaj, Omar K Abughanimeh, Anas Noman, Lara Kujtan

**Affiliations:** 1 Internal Medicine, University of Missouri-Kansas City School of Medicine, Kansas City, USA; 2 Hematology/Oncology, University of Nebraska Medical Center, Omaha, USA; 3 Internal Medicine, University of Missouri-Kansas City School of Medicine, Truman Medical Center, Kansas City, USA; 4 Hematology / Oncology, University of Missouri-Kansas City School of Medicine, Kansas City, USA

**Keywords:** rash, mixed cryoglobulinemia, prostatic cancer

## Abstract

Mixed cryoglobulinemia (MC) is well known for its association with chronic hepatitis C virus (HCV) infection. However, it has also been linked to autoimmune disorders and hematological malignancies, particularly of B-cell lymphoid origin. Association with solid malignancies is poorly described in the literature. Non-HCV-related MC, in the setting of prostate cancer, has been reported only twice. Here, we describe a case of MC in a prostate cancer patient complicated by membranoproliferative glomerulonephritis (MPGN) that responded well to plasma exchange therapy and treatment with both corticosteroids and rituximab.

## Introduction

Cryoglobulinemia is a state of precipitation of immunoglobulins (Igs) in a patient’s serum at temperatures lower than 37°C, which re-dissolve with rewarming [1]. Type I cryoglobulinemia refers to monoclonal Ig precipitation usually associated with hematological disorders, while types II and III refer to polyclonal composition with rheumatoid factor expression. Both type II and type III are also known as mixed cryoglobulinemia (MC). Cryoglobulinemia can present with arthralgia, fatigue, myalgia, neuropathy, as well as a vasculitic rash. Membranoproliferative glomerulonephritis (MPGN) is seen in 20 to 30% of cryoglobulinemic patients [2-4]. Here we present a case of MC complicated by MPGN in a patient without known history of hepatitis C virus (HCV) infection who was eventually diagnosed with prostatic cancer. To our knowledge, this association was reported only twice before.

## Case presentation

**Figure 1:**
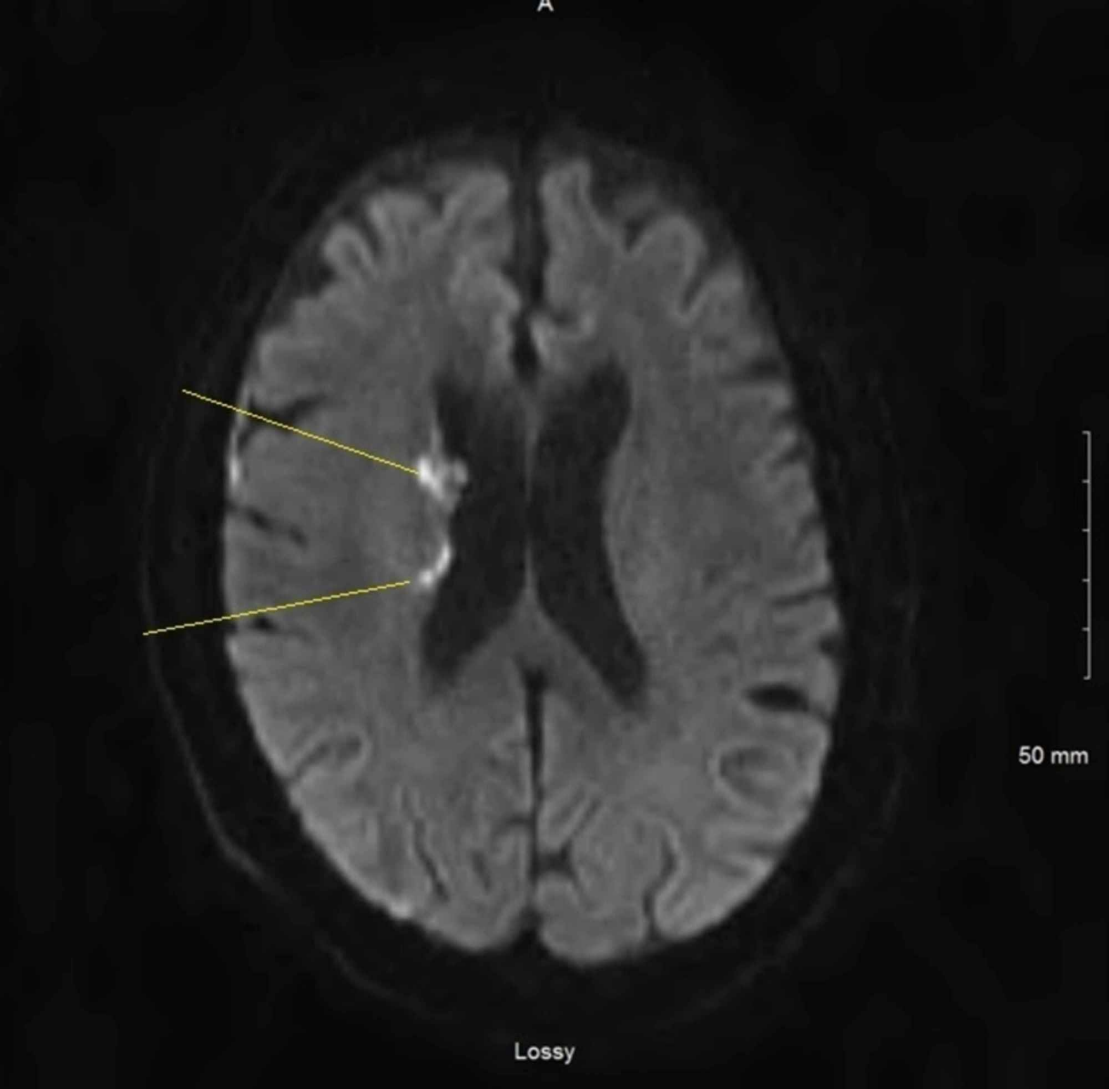
Magnetic resonance imaging of the brain showing patchy restricted diffusion and acute infarction.

A 66-year-old male with history significant for coronary artery disease, presented with an erythematous rash on his back, myalgia, fever, and confusion. On presentation, his temperature was 38.5°C, but the rest of vitals signs were normal. Labs were notable for creatinine of 1.4 mg/dL, alanine aminotransferase of 153 IU/L, aspartate aminotransferase of 207 IU/L, and lactate of 3.8 mmol/L. The patient was admitted for further workup and he was started on ceftriaxone and doxycycline to cover for meningitis and tick-borne illness respectively. Infectious disease team was consulted and he underwent extensive infectious evaluation which was negative for viral, bacterial or fungal infections. MRI of the brain revealed patchy restricted diffusion and acute infarction concerning for central nervous system (CNS) vasculitis (Figure 1).

Rheumatology team was consulted giving the MRI findings and autoimmune workup was sent including antinuclear antibodies, anti-double stranded DNA antibodies, serum protein electrophoresis, CH50, C3, C4, and cryoglobulin levels. In the following days, his creatinine got worse gradually reaching 5.8 mg/dL on hospital day four, so he was started on hemodialysis. By that time, his autoimmune workup was remarkable for positive cryoglobulin test, C3 of 70 mg/dl, C4 of <8 mg/dl, and the rest was negative. Testing for hepatitis C antibody and polymerase chain reaction were negatives. He was started on intravenous methylprednisolone 60 mg. The patient underwent kidney biopsy which showed acute interstitial nephritis and immune complex deposits suggestive of MPGN. The patient was transitioned to oral prednisone 60 mg daily, and underwent plasma exchange every other day for five sessions. Steroids were tapered and he was started on weekly rituximab for four weeks. His creatinine returned to baseline and hemodialysis was stopped. He was discharged later with a plan to follow up with his primary care physician (PCP) and rheumatologist.

One week after discharge, he followed up with his PCP who ordered prostate specific antigen (PSA) for prostate cancer screening. The level came back elevated of 22.60. He underwent prostate biopsy which showed prostatic adenocarcinoma with Gleason Score 3+4. Further workups including computed tomography of the abdomen/pelvis and bone scan were negative for metastatic disease. After discussing the options of treatment, he chose external beam radiation therapy.

## Discussion

Cryoglobulinemia is primarily diagnosed by immunoelectrophoresis of the cryoprecipitate after storing the serum sample at 4°C for eight days. A concentration above 50 mg/L (quantified using immunofixation or western blotting) is considered abnormal. Nonetheless, it has been noted that the serum concentration does not necessarily correlate with the severity of symptoms [5, 6].

It is well known that HCV infection is by far the most common cause of MC (90%) [7]. Other diseases associated with non-HCV MC include systemic lupus erythematosus, Sjogren’s syndrome and B-cell lymphomas [8]. Although a variety of body systems can be involved in MC resulting in different manifestations, renal involvement in non-HCV-infected patients is poorly described in the literature [9].

The association of cryoglobulinemia and solid tumors in non-HCV-infected patients remains rarely reported in the literature. Rullier et al. reported two patients with MC and solid tumors; one patient had breast cancer while the other presented with neoplasia of the bladder and lung [10]. To our knowledge, prostate cancer and non-infectious MC has only been reported twice, by Spatola et al. [11] and Milas-Ahić et al. [12]. However, in the latter case the patient was also treated for non-Hodgkin’s lymphoma and was diagnosed with gastric adenocarcinoma and Sjögren’s syndrome as well.

Of note, the patient reported by Spatola et al. [11] had renal involvement with MPGN, similar to our patient, and was only treated with corticosteroids. However, the patient failed to respond and became dependent on hemodialysis. Our patient responded well to corticosteroids, plasmapheresis, and rituximab therapy. This is in line with the ancillary study to the CryoVas survey, conducted by Zaidan et al. [4] which identified 80 patients with biopsy-proven noninfectious mixed cryoglobulinemic glomerulonephritis (MCGN) and concluded that rituximab plus corticosteroids prevented relapses more effectively compared to corticosteroids alone. However, this regimen resulted in a high rate of early death, usually associated with severe infections, when used as a first-line treatment. The authors concluded that further studies into decreasing the corticosteroid dose when used in combination are needed to determine whether the rate of severe infections improves [4].

## Conclusions

MC in non-HCV-infected patients is related to systemic diseases most of the time. Although the association of solid cancers and MC has rarely been described and the underlying pathogenesis remains unclear, we recommend to keep a potential malignant etiology on the differential. Furthermore, the usage of rituximab and corticosteroids in noninfectious MCGN appears to be efficacious in preventing relapses, with attention paid to the risk for severe infection.
